
JOURNAL INFORMATION
==============================
NLM Title Abbreviation: ScientificWorldJournal
Journal ID: ScientificWorldJournal
NLM Journal ID: 101131163
Journal ID: TSWJ

The Scientific World Journal

ISSN: 2356-6140
EISSN: 1537-744X
Publisher: Wiley

ARTICLE INFORMATION
==============================
PMCID: PMC5956360
PMID: 15349522
DOI: 10.1100/tsw.2004.40
Article ID: 159626
Article version: 1
Subjects: Case Report

Hematuria Due to an Intrapelvically Intruding Total Hip Replacement

Bach Christian M. 1 *
Steingruber Iris 2
Nogler Michael 1
Ogon Michael 1
Janetschek Günter 3
1 Department of Orthopedic Surgery, University of Innsbruck, Austria
2 Department of Radiology, University of Innsbruck, Austria
3 Department of Urology, University of Innsbruck, Austria
*Christian M. Bach: christian.bach@uibk.ac.atAcademic Editor: Anthony Atala

Publication date: 2004
Electronic publication date: 2004 Jun 7
Volume: 4
First page: 24
Last page: 26
Copyright: Copyright © 2004 Christian M. Bach et al.
Copyright year: 2004
License: This is an open access article distributed under the Creative Commons Attribution License, which permits unrestricted use, distribution, and reproduction in any medium, provided the original work is properly cited.
License URL: https://creativecommons.org/licenses/by/3.0/

==============================
Case Report
*TheScientificWorldJOURNAL* (2004) 4 (S1), 24–26
ISSN 1537-744X; DOI 10.1100/tsw.2004.40

### Hematuria Due to an Intrapelvically Intruding Total Hip Replacement

Christian Michael Bach*, MD[1], Iris Steingruber, MD[2], Michael Nogler, MD[1], Michael Ogon, MD[1], Günter Janetschek, MD[3]

[1]*Department of Orthopedic Surgery, University of Innsbruck, Austria;* [2]*Department of Radiology, University of Innsbruck, Austria;* [3]*Department of Urology, University of Innsbruck, Austria*

E-mails**:** **christian.bach@uibk.ac.at**

*Previously published in the Digital Urology Journal*

**DOMAIN**: urology

A 92 year old male patient was referred to our service because of gross hematuria. He had a history of numerous episodes of hematuria in the last year. A cystoscopy was performed as first measure. The bladder neck, the trigone and the ureteral orifices were normal. Inspection of the mucosa revealed a localized bleeding area three centimeter in diameter within the posterior wall of the bladder. This area was bulged by an extravesical solid tumor of unknown etiology. Local pressure had caused central necrosis, but no fistula could be observed.

Plain radiographs of the pelvis were performed revealing severe osteolytic destruction of the right acetabulum, a proximal fracture of the right superior pubic ramus and central transpelvic dislocation of the prosthetic stem of a total hip replacement with the femoral head supporting at the contralateral sacro-iliac joint (Figure 1). A computed tomography was performed demonstrating extravesical compression due to the dislocated prosthetic stem (Figure 3). Medical records revealed that the patient underwent total hip arthroplasty 22 years ago. Failure of the implant was reported but revision surgery was not performed. Revision surgery including removal of the intrapelvic prosthesis was initiated to avoid bladder rupture. Extraction of the stem was performed successfully, the patient recovered well and no further events of hematuria were recorded.

*Corresponding author: Department of Orthopedic Surgery, University of Innsbruck, Anichstrasse 35, A-6020 Innsbruck Austria; ++43-512-504-2690/Fax: ++43-512-504-2701

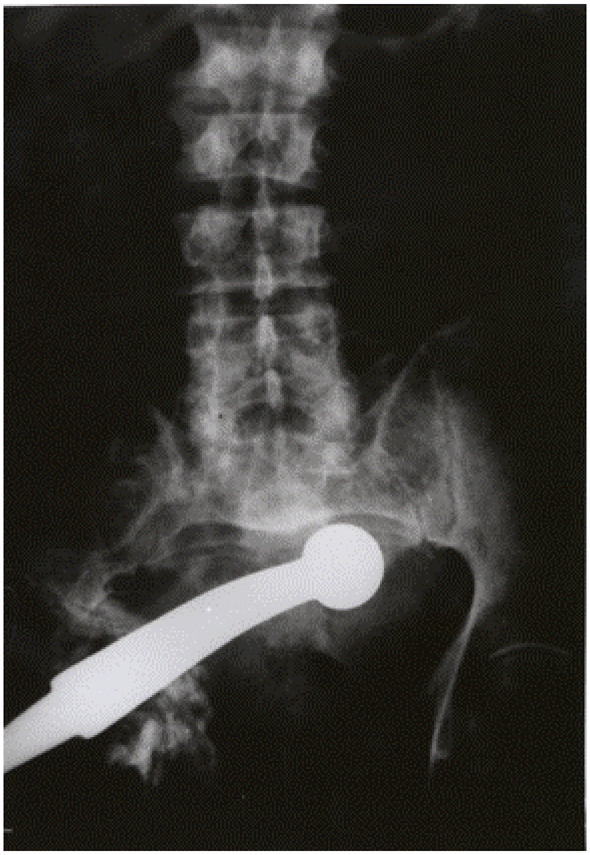

**Figure 1.** Plain radiograph of the pelvis with the transpelvically dislocted prosthetic stem (anterior-posterior view)

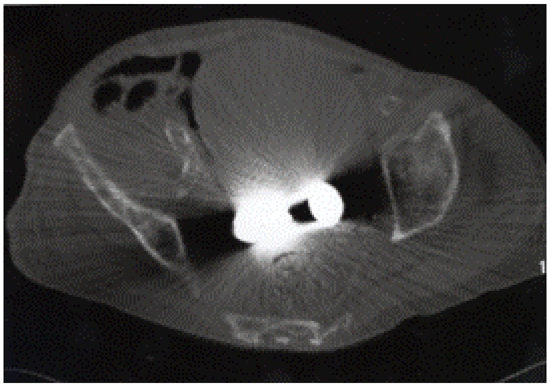

**Figure 2.** Computed tomography scans of the pelvis revealing the close proximity between the intrapelvically intruded prosthetic stem and the bladder (axial view)

### DISCUSSION

Hematuria is relatively common. Unless there is a known cause for hematuria, it must be thoroughly evaluated. Hematuria after routine hip surgery is very uncommon. Migration of a pin through the head of the femur into the adventitia of the left ureter1 and a penetrating bladder injury caused by a medially placed acetabular screw[2] were reported. Other uncommon reasons include vesico-cutaneous fistula,[3, 4, 5]

vesico-acetabular fistula,[5, 6, 7] trans-trochanteric vesico-cutaneous fistula[8] and fistula between the ureter and the acetabular space.9 Hematuria may also be due to either to the exothermic reaction involved in polymerization of the methylmethacrylate or the mass effect of the extruded intrapelvic cement or both.[10, 11, 12, 13]

Review of the literature disclosed no report of hematuria due to pressure of a dislocated prosthetic stem of a total hip replacement on the bladder. This mechanism must be considered when hematuria is detected in a patient with a medical history of total hip arthroplasty. We propose that a plain radiograph of the pelvis should be performed to exclude a lesion of the urinary tract by a dislocated arthropla orthoplasty.

### LITERATURE

1. Kleinman, S.Z. and Russin, L.A. : Migration of left hip pin with involvement of left ureter. Urol., **16**: 396, 1980.
2. Kinmont, J.C. : Penetrating bladder injury caused by a medially placed acetabular screw. J South Orthop Assoc., **8**: 98, 1999.
3. Gallmetzer, J., Gozzi, C. and Herms, A. : Vesicocutaneous Fistula 23 Years after Hip Arthroplasty. A case report. Urol Int., **62**: 180, 1999.
4. Memon, F.R., Foss, M.V. and Towler, J.M. : Haematuria and vesico-cutaneous fistula after hip surgery. Br J Urol., **79**: 1005, 1997.
5. Hamano, S., Tanaka, M., Igarashi, T., Kojima, S., Suzuki, N. and Murakami S. : Vesico-acetabular fistula occurring 10 years after total hip replacement. Hinyokika Kiyo., **42**: 743, 1996.
6. Schneider, H.J. and Mufti, G.R. : Haematuria and vesicocutaneous fistula after hip surgery. Br J Urol., **80**: 971, 1997.
7. Tripp, B.M., Tanzer, M., Laplante, M.P. and Elhilali, M.M. : Vesico-acetabular fistula. J Urol., **153**: 1910, 1995.
8. Tremeaux, J.C., Laloux, E., Striffling, V. and Briet, S. : Trans-trochanteric vesico-cutaneous fistula after total hip replacement. Ann Urol. (Paris), **23:** 123, 1989.
9. Schafer, D., Mattarelli, G. and Morscher, E. : Ureteroarticular fistula after total hip replacement. A case report. Arch Orthop Trauma Surg., **114:** 35, 1994.
10. Greenspan, A. and Norman, A. : Gross hematuria: a complication of intrapelvic cement intrusion in total hip replacement. Am J Roentgenol., **130**: 327, 1978
11. McCallum, T.J., O´Connor, G.J. and Allard, M.J. : Intravesical methylmethacrylate after revision hip arthroplasty. J Urol., **156**: 1777, 1996.
12. Ray, B., Baron, T.E. and Bombeck, C.T.: Bladder and ureteral diaplacement complication of total replacement hip arthroplasty. Urol., **13**: 554, 1979.
13. Wheeler, J.S., Babayan, R.K., Austen, G. Jr. and Krane, R.J.: Urologic complication of hip arthroplasty. Urol., **22**: 499, 1983.

**This article should be referenced as follows:**

Bach, Christian Michael; Steingruber, Iris; Nogler, Michael; Ogon, Michael; Janetschek, Günter (2004) Hematuria Due to an Intrapelvically Intruding Total Hip Replacement *TheScientificWorldJOURNAL* **4 (S1)**, 24–26.

**Handling Editor:**

Anthony Atala, Principle Editor for *Urology* — a domain of *TheScientificWorldJOURNAL*.
